# “I'M NOT A RISK-TAKER”: RISK PERCEPTIONS OF NURSING HOME RESIDENTS WITH DEMENTIA

**DOI:** 10.1093/geroni/igac059.918

**Published:** 2022-12-20

**Authors:** Liza Behrens, Hannah Anderson, Kalei Kowalchik, Jacqueline Mogle, Kimberly Van Haitsma, Marie Boltz

**Affiliations:** Pennsylvania State University, University Park, Pennsylvania, United States; Children's Hospital of Philadelphia, Philadelphia, Pennsylvania, United States; Pennsylvania State University, University Park, Pennsylvania, United States; The Pennsylvania State University, University Park, Pennsylvania, United States; Pennsylvania State University, University Park, Pennsylvania, United States; Penn State, Pennsylvania State University, Pennsylvania, United States

## Abstract

Persons living with dementia (PLWD) in nursing homes (NH) are often left out of care conversations about their health and safety. These omissions impinge on their personhood and rights to have care preferences heard and honored. PLWD maintain the ability to communicate values and preferences long after their decision-making abilities are affected by cognitive changes. This study explored risk perceptions of PLWD associated with their care preferences. As part of a larger focused ethnography conducted during the COVID-19 pandemic, in-depth interviews explored risk perceptions of residents (N=7) with dementia (BIMS M=9.29). Using a risk propensity survey, residents self-identified as risk avoiders (M=3.2) and content analysis of interviews revealed that PLWD perceive physical and psychosocial harms (e.g., high blood sugar, falls, choking) and benefits (e.g., feeling good, social interactions, reminiscing) related to care preferences. Results suggest it is possible for PLWD with varying levels of cognitive decline to participate in discussions about their health and safety.

